# Axon Terminal Arbors of Retinal Horizontal Cells Lose Control

**DOI:** 10.3389/fncir.2018.00082

**Published:** 2018-10-11

**Authors:** Benjamin E. Reese

**Affiliations:** Department of Psychological and Brain Sciences, Neuroscience Research Institute, University of California, Santa Barbara, Santa Barbara, CA, United States

**Keywords:** Netrin-G ligand 2, rod spherule, outer plexiform layer, coverage factor, synaptogenesis

The dendritic arbors of the post-receptoral neurons in the retina, the horizontal cells and bipolar cells, spread their processes during development in a manner that establishes a uniformity in their coverage across the retinal surface, yet the degree of dendritic overlap varies conspicuously (Reese and Keeley, 2015). Their developmental plasticity has been most extensively studied in the mouse retina, where each type of cone bipolar cell (CBC) establishes a dendritic coverage of 1 (being the average number of dendritic fields overlying each location across the retina), extending their dendritic arbors to the margins of their immediate neighbors, thereby tiling the retina (Wässle et al., 2009). Individual cone pedicles are consequently connected to just one of each type of CBC, although those at the borders of two like-type dendritic fields may connect to both. Rod bipolar cells (RBCs) establish a dendritic coverage factor of ~2–3, with each rod spherule contacting on average two RBCs (Tsukamoto and Omi, 2013), and with each RBC receiving input from ~90% of the rods within its field (Johnson et al., 2017). The dendritic arbors of horizontal cells (HCs), by contrast, overlap far more extensively, reaching a dendritic coverage factor of about ~6–7 (Reese et al., 2005). Their dendritic arbors contact all pedicles within a field, although colonization of those pedicles declines as a function of distance from the HC soma, where other more closely positioned HCs colonize a greater share of each pedicle's territory.

The uniformity in this coverage, regardless of cell type, is thought to arise from these cells being constrained in their dendritic growth by the presence of their homotypic neighbors, and while the experimental evidence for this is limited to just a few cell types, it likely holds for all of them: when cell density is experimentally increased or decreased, so dendritic field extent shows a corresponding decline or expansion, respectively (Poché et al., 2008; Lee et al., 2011; Reese et al., 2011; Johnson et al., 2017). While this might seem like a sensible way to organize every type of retinal mosaic, not all retinal cell types behave so, but to date, those exceptions are cell types with processes in the inner plexiform layer (Reese and Keeley, 2015). How such homotypic interactions rein in further territorial expansion is still unclear, but may involve contact-mediated repulsion at the dendrites (the CBCs; Lee et al., 2011), or by dendritic contact with neighboring somata (the HCs; Reese et al., 2005), or perhaps through a competition for the colonization of presynaptic partners (the RBCs).

What remains unaddressed, in the outer plexiform layer, is the behavior of the axon terminal arbor of the HC. In the mouse retina, there is only a single type of HC (Peichl and González-Soriano, 1994), where the dendritic arbor subserves cone photoreceptor circuitry while the axon terminal arbor is dedicated to rod circuitry. The axon terminal arbor emerges from a long process that extends away from the soma, serving a large and often irregular field of rod photoreceptors, where as many as 10 arbors overlie one another. Unlike the dendritic arbor, however, the axon terminal arbor contacts only a minority of the rod spherules within its field, but detailed quantitative studies in the mouse retina have yet to be conducted. In cat and primate retina, more than a single axon terminal ending is found in each spherule (Migdale et al., 2003), but the number of axon terminals contributing them remains to be determined. In the rabbit retina, each rod spherule accommodates only a single axon terminal ending, and each terminal arbor contacts about a tenth of the local rod spherule population, apportioning them selectively amongst the collection of 10 overlapping arbors (Pan and Massey, 2007). Whether the area of the arbor is constrained by the local density of homotypic arbors, and how that arbor chooses amongst the collection of spherules within its catchment area for establishing synaptic contacts, is unknown.

The axons of HCs have a long history of behaving in unexpected ways. Notably, they are prone to sprouting well after they have established their laminar organization and synaptic connectivity during development, exhibiting a reactivity to multiple forms of insult by extending processes into the inner or outer nuclear layers and sub-retinal space (Peichl and Bolz, 1984; Fisher et al., 2005; Specht et al., 2007; Nagar et al., 2009). This capacity for reentering an active growth-mode in maturity may not prove unique to the HCs; indeed, RBCs exhibit a capacity for sprouting after they have established their laminar organization that is normally held in check by the HCs themselves (Keeley et al., 2013). But a new study demonstrates the molecular control of the size and connectivity of the axon terminal arbor, including its capacity for plasticity in the mature retina.

Soto et al. (2013) previously demonstrated that the synaptic cell adhesion molecule Netrin-G ligand 2 (NGL2) plays a role in establishing these features of the axon terminal arbor. Only HCs express NGL2 in the outer retina, yet they target the protein exclusively to the terminal endings of the arbor, at the sites where these form contacts with rod spherules. The photoreceptor population expresses Netrin-G2, being the likely synaptic partner associated with NGL2 at the tips of the arbor. In *Ngl2* knockout mice, Soto et al. (2013) observe three striking features: first, the HCs sprout processes into the outer nuclear layer; second, they expand the area of their axon terminal arbor by 61% (without any effect upon the dendritic arbor); and third, the number of terminal endings per unit area of the arbor field declines by 58%. Because HC densities are not altered in these retinas, the axon terminal arbors are not expanding due to any reduction in homotypic neighbors, and so the coverage factor of these arbors must increase, from ~10 in the wild-type retina to ~16 in the *Ngl2* knockout retina (Soto et al., 2013). These results are intriguing because they show that, rather than modulate areal size in relation to the presence of homotypic neighbors in order to maintain a constant coverage, the size of the arbor is controlled by its relationship with the population of rod spherules. That arbor size might be influenced by afferent density was previously shown in a study comparing the size of the arbor in rod-full vs. cone-full mutant retinas (Raven et al., 2007). The study by Soto et al. (2013) convincingly demonstrates a molecular basis for an interaction with the photoreceptors.

Since then, Soto and coworkers have extended this research to show that these effects are identical when they eliminate *Ngl2* in single HCs using an AAV-mediated CRISPR-Cas9 strategy (to express a short guide RNA targeting *Ngl2* in mice ubiquitously expressing Cas9). Furthermore, they show that these effects are not limited to the developmental window when HCs are first establishing the laminar and areal distributions of their axon terminal arbors, but can be achieved, to a comparable magnitude, even when the AAV-mediated knockout is administered as late as 5 months of age (Soto et al., 2018). Finally, they go on to show, by way of AAV-mediated overexpression of *Ngl2* in HCs in the *Ngl2* knockout mouse, that they can recover their wild-type features, by reducing arbor area and increasing terminal ending density, both to apparently normal values. These results resolve one issue from the former study, that the effects produced by manipulating NGL2 are indeed cell-autonomous. More critically, they show that NGL2 would appear to mediate a tonic constraint upon the size of the axon terminal arbor throughout life. Deprived of this influence, the arbor re-enters an expansionist growth mode, as if it is seeking prospective synaptic partners when the contacts that it already has are now destabilized.

These results consequently raise interesting questions about the relationship between synapse formation at the rod spherule and the size of the axon terminal arbor during development. The authors show that *Ngl2* expression in HCs is discrete at postnatal day 10, yet protein is not conspicuous in the outer plexiform layer until sometime later, around P15-20. As axon terminal arbors have commenced formation well before this stage (Raven et al., 2007), the onset of NGL2 in the population of overlapping arbors may trigger the colonization of spherules and/or the stabilization of synaptic contacts therein, in turn triggering a cessation of further territorial expansion. But how overlapping arbors might relate to these processes of spherule colonization and arbor areal growth remains to be unraveled.

Soto et al. (2018) report the frequent colocalization of terminal endings at individual spherules from two different overlapping arbors. Yet these authors had formerly reported that the number of terminals per arbor averages around 400 (Soto et al., 2013). Given that the number of HCs in the C57BL/6J retina is ~18,500 (Whitney et al., 2011), this yields an estimate of the total number of terminal endings made by the entire population of HCs to be about 7,400,000, close to the estimated size of the total rod photoreceptor population in this same strain, being about 7,624,000 (Keeley et al., 2014). While a clear resolution of this issue may require EM reconstructions of multiple spherules, the above calculations suggest that rod spherules in the mouse, like those in the rabbit, are connected to only a single axon terminal arbor. Such a conclusion becomes particularly interesting in light of Soto et al.'s (2018) results overexpressing *Ngl2* in single axon terminal arbors in the wild-type retina. There, they report an increase in the density of terminal endings, suggesting that overexpressing arbors, amidst a sea of normally expressing arbors, may actually displace terminal endings from those wild-type arbors.

NGL2 may therefore play a role in promoting the successful colonization of rod spherules, and coupled with a spherule-intrinsic constraint of accommodating only a single terminal ending, overlapping arbors would normally apportion the population of spherules stochastically. While terminal ending density and arbor size are clearly inversely related, the axon terminal arbor is not specified to make a particular number of terminal endings, as the total number per arbor is also reduced, dropping from about 400 per arbor to 300 in the *Ngl2* knockout retina. Coincident with that decline, so the authors report an increase in the proportion of spherules lacking *any* axon terminal endings in electron micrographs from the *Ngl2* knockout retina (Soto et al., 2013).

What seems clear is that lacking the normal onset of NGL2 function during development, these arbors are compromised in stabilizing spherule contacts, and consequently continue to expand their areal extents until they reach an upper limit, perhaps reflecting an intrinsic constraint on their maximal size. So while having NGL2 is important, both for making a normal-sized arbor and for connecting with a normal number of rod spherules, its absence doesn't yield a completely out-of-control arbor nor produce a total loss of invaginating endings from the population of spherules. Consistent with those results, the dark-adapted b-wave of the ERG remains intact, if slightly reduced in magnitude in the *Ngl2* knockout mouse (Soto et al., 2013). But why the areal size of the axon terminal arbor and the density of terminal endings should both revert to those seen in wildtype retina when *Ngl2* function is restored to individual cells in the knockout retina, given the absence of NGL2 in most of the overlapping axon terminal arbors (Soto et al., 2018), is not readily explained by any interpretation that credits homotypic interactions with sculpting final connectivity. Further studies should clarify whether imbalancing NGL2 in terminal arbors, rather than completely eliminating it, proves most illuminating for our understanding of its role during normal development and in maturity.

The authors acknowledge the possibility that NGL2 may be playing independent roles in these two processes (perhaps even three if one also considers the sprouting phenotype observed in both studies, but left relatively unaddressed). Whether homotypic neighbors play any direct role in constraining one another's colonization of spherules remains to be tested directly. Resolving that issue may shed light on the relationship between arbor size and innervation density, and potentially clarify the role of NGL2 in this relationship.

## Author contributions

The author confirms being the sole contributor of this work and has approved it for publication.

### Conflict of interest statement

The author declares that the research was conducted in the absence of any commercial or financial relationships that could be construed as a potential conflict of interest. The reviewer AK and handling editor declared their shared affiliation at time of review.
